# Pregnancy related acute kidney injury: A single center experience from the Kashmir Valley

**DOI:** 10.4103/0971-4065.45291

**Published:** 2008-10

**Authors:** M. Saleem Najar, A. Rashid Shah, I. A. Wani, A. Rashid Reshi, K. A. Banday, M. Ashraf Bhat, C. L. Saldanha

**Affiliations:** Departments of Nephrology, and Gynaecology, Sher-i-Kashmir Institute of Medical Sciences, Srinagar, J & K, India; 1 Department of Obstetrics, Sher-i-Kashmir Institute of Medical Sciences, Srinagar, J & K, India

**Keywords:** Acute kidney injury, mortality, pregnancy, septic abortion

## Abstract

All patients admitted with pregnancy related acute renal failure (PRAKI) from June 2005 to May 2007 were studied with respect to etiology, clinical features, and outcome of PRAKI. Of 569 cases of acute kidney injury (AKI), 40 (7.02%) cases were related to gestational problems; the age of the patients ranged from 15 to 45 years. Septic abortion was the most common cause of PRAKI, accounting for 20 (50%) cases of which 15 (75%) cases occurred in the first and five (25%) in the second trimester. Other causes were antepartum hemorrhage: six cases (15%), toxemia of pregnancy: six cases (15%), acute gastroenteritis: three cases (7.5%), postpartum hemorrhage: two cases (5%), acute pyelonephritis: two cases (5%), and postpartum, acute kidney injury: one case (2.5%). Dialysis was needed in 60% of the cases and mortality was observed in 20% of the cases. PRAKI continues to be a major concern in our society, causing a high maternal mortality. Septic abortion which has virtually disappeared from developed countries, continues to be a major cause of PRAKI in our society. Hence, there is a need to halt the practice of illegal abortions and improve antenatal care.

## Introduction

Acute kidney injury (AKI) is a rare but life-threatening complication of pregnancy. The incidence of AKI has sharply declined from 0.5 per 1000 pregnancies to one in 20,000 births in developed countries.^1^ No case of AKI was observed in 12,000 and 20,000 births in two series.^2^,^3^ On the other hand, pregnancy is still responsible for 15–20% of AKI in developing countries.^4^,^5^ Pregnancy related AKI (PRAKI) is on the decline from 14.5% reported in 1987 to 4.3% in 2005 in India.^6^,^7^ Septic abortion is the most common cause of AKI in early pregnancy, whereas toxemia of pregnancy, hemorrhage, and ischemic, acute, tubular necrosis occur in late pregnancy.^1^,^8^ Rare causes of PRAKI include acute fatty liver, HELLP Syndrome in the third trimester of pregnancy, and puerperal sepsis and thrombotic microangiopathy in the postpartum period. Septic abortion is the most common cause of PRAKI in developing countries,^6^,^8^ but its worldwide incidence has declined significantly.^9^,^10^ We present here our experience with PRAKI from the Kashmir Valley; our institute located in Srinagar is the only tertiary care center with a dialysis facility in the valley of Kashmir.

## Materials and Methods

Of 569 AKI patients admitted to the Nephrology Department of our institute from June 2005 to May 2007, 40 (7.02%) were associated with pregnancy. The causes of AKI, its clinical features, need for dialysis, and the outcome were examined prospectively. Data on the age of patients, number of pregnancies, history of previous renal disease or hypertension, and prior births were noted. The results of a detailed medical history, physical examination, routine urine analysis, levels of blood urea nitrogen and serum creatinine, fractional excretion of sodium, and renal ultrasonography were noted.

PRAKI was diagnosed when there was sudden-onset oliguria (urine output < 400 mL in 24 hours) or anuria with serum creatinine elevated to > 1.5 mg%. Patients with underlying chronic kidney disease were excluded from the study.

The data were analyzed using SPSS software and the results were recorded as median ± standard deviation (SD). Chi square and Fisher's exact tests were used.

## Results

Of the 569 cases of acute kidney injury (AKI), 40 (7.02%) cases were related to gestational problems. The age of patients ranged from 15 to 45 years with a mean of 28.94 ± 5.93 years. Twelve (30%) patients were primigravid and 28 (70%) were multigravid in our study.

The causes of pregnancy-related, acute kidney injury (PRAKI) are shown in Table 1. Septic abortion was the most common cause accounting for 20 (50%) of the women with PRAKI, 15 (75%) of which occurred in the first trimester and five (25%) in the second trimester. Of 20 patients with septic abortion, 18 (90%) were from rural areas and their abortions had been conducted by untrained midwives.

**Table 1:** Causes of pregnancy-related AKI (n=40)

Causes	Number	Percent
Septic abortion	20	50
Antepartum hemorrhage	6	15
Toxemia of pregnancy	6	15
Acute gastroenteritis	3	7.5
Postpartum hemorrhage	2	5
Acute pyelonephritis	2	5
Postpartum	1	2.5

AKI: Acute kidney injury

The symptoms and signs at the time of admission are shown in Table 2. Oliguria was present in all patients and the average hospital stay was 1–26 days (1.39 ± 6.29). The laboratory values at the time of admission are given in Table 3. Anemia (Hb < 10 gm/dL) was seen in 32 (80%) patients, hyperkalemia in 12 (30%) cases, leucocytosis in ten (25%), hyponatremia in six (15%), and hypoalbuminemia in six (15%) patients.

**Table 2:** Symptoms and signs (n=40)

Parameter	Number	Percentage
Oliguria	40	100
Fever (Temp > 37°C)	32	80
Edema	24	60
Shortness of breath	20	50
Encephalopathy	15	37.5
Hypotension (90/60 mmHg)	10	25
Hypertension (140/90 mmHg)	7	17.5
Convulsions	4	10

**Table 3:** Laboratory results

Laboratory values	Mean ± SD
Hemoglobin (g/dL)	7.01 ± 2.56
Serum urea (mg/dL)	206.46 ± 95.46
Serum creatinine (mg/dL)	7.2 ± 3.5
Serum albumin (g/dL)	2.63 ± 0.76

Pregnancies were terminated by cesarean section in six (15%) patients and by induction in six other (15%) cases. Hysterectomy was needed in nine (22.5%) cases and repair of cervical tear was required in one (2.5%) case. ARDS developed in ten (25%) cases, pneumonia in five (12.5%) cases, disseminated intravascular coagulation in four (10%) cases, and suppurative cholangitis in one (2.5%) case.

Hemodialysis was given to 13 (32.5%) cases, peritoneal dialysis to six (15%) cases and both modalities to five (12.5%) cases, whereas only medical treatment was given to 16 (40%) patients. Mortality was observed in eight (20%) cases. The causes of death are given in Table 4. Twenty-nine (72.5%) patients recovered completely, two (5%) showed partial recovery, and one (2.5%) patient remained dependent on dialysis. Bilateral renal cortical necrosis was documented in a contrast-enhanced CT scan in this patient who presented with anuria and remained dependent on dialysis.

**Table 4:** Causes of death (n=8)

Cause of death	Number	Percentage
ARDS	5	62.5
Septic shock with DIC	2	25.0
GI bleeding	1	12.5

## Discussion

Pregnancy-related, acute kidney injury (PRAKI) is a rare entity in the West but continues to be a major problem in developing countries, resulting in a high maternal mortality. The frequency distribution of PRAKI is bimodal in relation to the period of gestation.^11^,^12^ The first peak is seen between seven and 16 weeks, mainly due to septic abortion, while toxemia of pregnancy, hemorrhage, and puerperal sepsis account for the second peak which is seen between 34 and 36 weeks.^1^,^6^

The worldwide incidence of PRAKI has deceased markedly in the past 50 years from 20 to 40% in the 1960s to < 10% in more recent series, largely due to the legalization of abortion and improved antenatal and obstetric care. No case of PRAKI was observed in 12000 and 20000 live births in two recent studies.^3^,^13^

Recent epidemiological studies have also confirmed the decreasing incidence of PRAKI in India, with a decrease from 14.5% in 1987 to 4.3% in 2005.^6^,^7^ Frequency of PRAKI reported in India is shown in Table 5. This too is due to the legalization of abortion and better antenatal care.

**Table 5:** Frequency of PRAKI reported in India

Author (year)	Number	PRAKI as % of total AKI
Pandith (1986 unpublished data)^14^	64	06
Chugh (1987)^6^	1862	14.5
Prakash *et al*. (1995)^8^	59	13.9
Rani *et al*. (2002)^9^	82	12.2
Kilari *et al*. (2006)^7^	41	04.3
Present study	40	07

PRAKI: Pregancy-related acute kidney injury, AKI: Acute kidney injury

There are a few studies from the Kashmir Valley that address the issue of PRAKI. Pandith *et al*.^14^ reported the incidence of PRAKI as 6% in the Kashmir Valley (unpublished data), whereas the incidence of PRAKI was 7% in our study. Septic abortion was the main cause of PRAKI in our series accounting for 20 (50%) cases, mostly conducted by untrained personnel (midwives and *dais*), eight hemorrhage (20%) cases and six toxemia (15%) cases were other common causes of PRAKI.

Although there has been a significant decline in PRAKI at the international and national levels, it continues to be static in the Kashmir Valley, largely due to an insignificant decline in septic abortion. Hence, there is a need for education and improvement in ante- and postnatal care, especially in the rural areas, and the practice of illegal abortions by untrained personnel has to be stopped.

The mortality related to PRAKI has declined to < 10% in Europe and North America,^1^ while the reported mortality rate of PRAKI has decreased from 56% in 1987 to 24.39% in 2005 in India.^6^,^7^

The mortality rate was 20% in our study, which is in accordance to current trends in India but still significantly higher compared to the developed countries (Table 5).

## Conclusion

PRAKI continues to be of significant occurrence accounting for 7% of AKI in our study, resulting in high maternal mortality. Septic abortion was the most common etiological factor responsible for 20 (50%) cases. Although there has been a significant decline in PRAKI at the international and national levels, it continues to be static in the Kashmir Valley due to an insignificant decline in septic abortion. Hence, there is a ,need to improve antenatal care particularly in rural areas, and the practice of illegal abortions by untrained personnel has to be stopped.
